# Guidance for Demonstrating the Societal Value of new Antibiotics

**DOI:** 10.3389/fphar.2020.618238

**Published:** 2021-02-01

**Authors:** Steven Simoens, Isabel Spriet

**Affiliations:** ^1^ KU Leuven Department of Pharmaceutical and Pharmacological Sciences, Leuven, Belgium; ^2^ Pharmacy Department, University Hospitals Leuven, Leuven, Belgium

**Keywords:** antibiotic, value, economic evaluation, transmission, resistance

## Abstract

Given that antibiotic use is associated with externalities, standard economic evaluation which considers costs and health gains accruing to patients under-values antibiotics. Informed by a scoping review, this discussion paper aims to identify the societal value elements of antibiotics and to provide guidance on how these value elements can be incorporated in economic evaluation. With a view to appropriately quantify the societal value of antibiotics, there is a need for good practice guidelines on the methodology of economic evaluation for such products. We argue that it is important to assess antibiotics at population level to account for their transmission, diversity, insurance, spectrum, novel action and enablement values. In addition to the value of antibiotics to infected patients, economic evaluations need to use modeling approaches to explore the impact of different modes of employing new and existing antibiotics (for example, as last resort treatment) on disease transmission and resistance development in current and future patients. Hence, assessing the value of antibiotics also involves an ethical dimension. Further work is required about how the multiple value elements of antibiotics are linked to each other and how they can be aggregated.

## Introduction

In recent years, stakeholders such as the International Society for Pharmacoeconomics and Outcomes Research (ISPOR), the European Society for Medical Oncology (ESMO) and the Institute for Clinical and Economic Review (ICER) have distinguished between multiple value elements of health technologies (Neumann et al., 2018). This builds further on standard economic evaluation, in which the value of a health technology in essence is captured in terms of the costs and health gains that accrue to patients. However, it has been argued that such a perspective is too narrow, especially in the case of new antibiotics where additional elements contribute to the value of these medicines (Karlsberg Schaffer et al., 2017). If such elements are not taken into account in economic evaluation, there is a risk that antibiotics are under-valued, that the decision to reimburse an antibiotic based on its value is misinformed and that pharmaceutical companies do not receive an appropriate reward for their antibiotic research and development efforts.

To date, little attention has been paid to identifying, measuring and quantifying the value elements of antibiotics. Also, reimbursement agencies do not have separate methodological guidelines pertaining to the economic evaluation of antibiotics. Furthermore, the experience with antibiotic assessment practices in Europe indicates that the different value elements of antibiotics are not taken into account systematically by reimbursement agencies and, at best, are considered in a qualitative manner. For instance, surveys have shown that the impact on antimicrobial resistance has been mentioned or implied in economic evaluations of individual antibiotics submitted to reimbursement agencies, but this and other values were not incorporated in a structured way in the ultimate decisions (Charafi and Chen, 2017; Morton et al., 2019).

As part of the EU One Health Action Plan against Antimicrobial Resistance, the European Commission highlighted the need to further develop the methodology for economic evaluation of antibiotics (European Commission, 2017). Therefore, the aim of this discussion paper is to identify the value elements of antibiotics and to discuss potential methodological approaches to incorporate these value elements in the economic evaluation framework. These issues are illustrated by referring to published economic evaluations of new antibiotics. Based on the results, recommendations are provided about how to assess the societal value of antibiotics. A priori, this paper assumes that antibiotics are prescribed in a rational and cost-effective way in accordance with clinical guidelines, and does not consider self-medication which may undermine the value of antibiotics.

This discussion paper was informed by a scoping review (Peterson et al., 2017), because this methodology is particularly suited to provide a narrative synthesis of an emerging field of research such as that related to the value assessment of antibiotics, to consider both peer-reviewed and gray literature, to identify knowledge gaps, and to propose avenues for future research, policy and practice. The scoping review was carried out according to the framework proposed by Arksey and O'Malley (2005) (Arksey and O'Malley, 2005). The literature search in PubMed and Google Scholar used a combination of search terms related to antibiotics (i.e., “antibiotic”, “infection”, “infectious disease”, “resistance”), economic evaluation (i.e., “economic evaluation”, “health technology assessment”, “cost-effectiveness”, “value”), and value elements (i.e., “unmet medical need”, “transmission value”, “diversity value”, “insurance value”, “spectrum value”, “novel action value”, “enablement value”). The bibliography of included articles and ISPOR conference abstracts were also searched for relevant material. There were no restrictions on the publication year or design of studies, but only English-language articles were considered. Economic evaluations of new antibiotics were included if they assessed value elements in addition to standard cost-effectiveness. To the extent that this is relevant to the economic evaluation of antibiotics, the review also drew on the value assessment of other types of medicines that have specific characteristics, such as vaccines, or treatments for other multi-drug resistant diseases, such as tuberculosis. Articles that discussed challenges in research and development of antibiotics, that explored the antibiotic pipeline, or that examined payment/reimbursement models for antibiotics were excluded.

**FIGURE 1:**
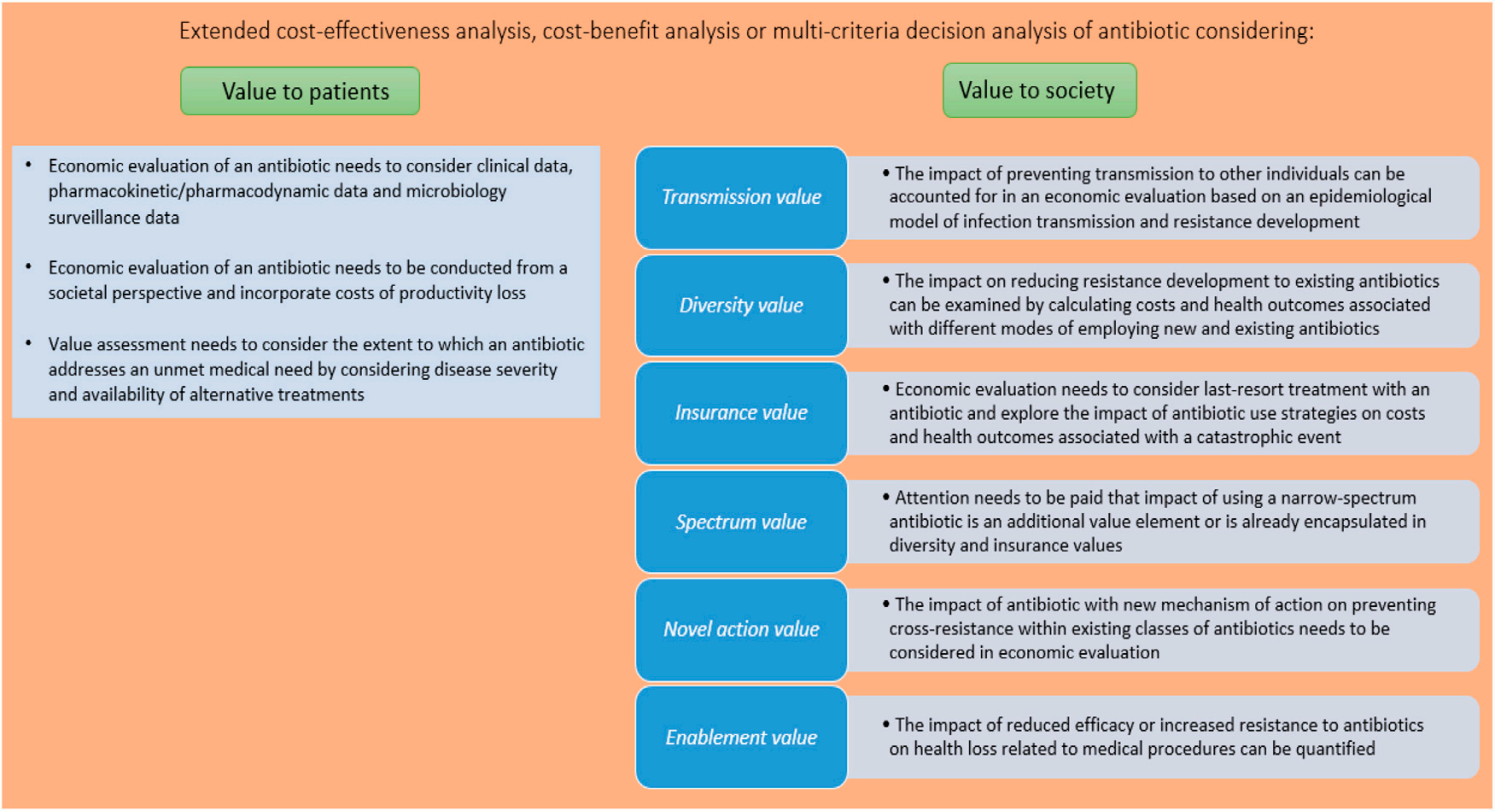
Assessing the value of antibiotics.

Figure 1 identifies and provides guidance on how the different value elements of antibiotics can be quantified and aggregated in the economic evaluation framework. Each value element is discussed in more detail in the following sections.

### Value Accruing to Patients

A standard economic evaluation of an antibiotic compares the health care (and other) costs and health gains (measured by, for example, clinical cure rates or quality-adjusted life years (QALYs)) for patients treated with that antibiotic as compared to the standard of care. However, there are challenges involved in such an exercise, particularly with respect to outcomes. It is difficult to consider the health gain of an antibiotic in an economic evaluation when the outcome of antibiotic therapy is typically derived from a non-inferiority trial. Therefore, marketing authorization of antibiotics is informed not only by such clinical data, but also by pharmacokinetic/pharmacodynamic (PK/PD) data and microbiology surveillance data. As regulators such as the European Medicines Agency have increasing experience with assessing these clinical and non-clinical data, they could educate reimbursement agencies on how to use such data in the context of an economic evaluation of an antibiotic. For instance, Rothery et al. illustrated how antibiotic efficacy could be quantified by means of clinical success rates, microbiological eradication rates and resistance rates in an economic evaluation focusing on *Acinetobacter baumannii* infections (Rothery et al., 2018). Given that microbiology surveillance data can be seen as a type of real-world evidence (RWE) (Rothery et al., 2018), agencies can also draw on guidelines on the use of RWE for reimbursement decision making (Garrison et al., 2007). A second challenge related to outcomes is that existing methods to elicit the disutility of acute infection (in the context of calculating the effectiveness of an antibiotic in terms of quality-adjusted life years) are not particularly suited to transient infectious diseases (Bala and Zarkin, 2000). Therefore, the use of alternative outcome measures such as disability-adjusted life years (DALYs) and willingness to pay has been advocated, even though these measures also suffer from limitations (Holmes and Hughes, 2019).

While the scope of costs considered in an economic evaluation of an antibiotic is restricted to health care costs in Belgium, reimbursement agencies in for example the Netherlands and Sweden adopt a broader perspective and also include costs of productivity losses. These costs can be substantial in the context of infectious diseases: the World Bank estimated that the macro-economic impact of worker morbidity and mortality stemming from antimicrobial resistance could reduce global gross domestic product by 1.1%–3.8% by 2050, which is of the same order of magnitude as the impact of the 2008–2009 global financial crisis (Jonas et al., 2017). Consideration of productivity losses improves the value of an antibiotic as exemplified by an Italian economic evaluation which showed that the incremental cost-effectiveness ratio of bedaquiline plus background drug regimens for resistant tuberculosis fell from €16,639 per life year gained to €4,081 per life year gained (Codecasa et al., 2017).

It could be argued that an antibiotic is more valuable if it addresses an unmet medical need. Unmet medical need relates to the severity of the disease and the availability of alternative treatments, two facets which specifically apply to treatment of multi-drug resistant infections with antibiotics (Karlsberg Schaffer et al., 2017). Even though unmet medical need can be appraised by referring to, for example, the World Health Organization Priority Pathogens List (World Health Organisation, 2017), the literature does not elucidate how this can be taken into account in the value assessment of an antibiotic. Reimbursement agencies tend to consider unmet medical need in their decision making process (Vreman et al., 2019): for instance, *the importance of the medicine in clinical practice as a function of therapeutic and social needs* is one of the criteria evaluated by the Belgian Drug Reimbursement Committee (Koninklijk Besluit van, 2018). Also, a medicine can be reimbursed (even prior to marketing authorization) in Belgium if it tackles a disease included in the National Institute for Health and Disability Insurance’s list of unmet medical needs. In 2020, treatment of *Clostridioides difficile* infections is on this list (RIZIV-INAMI, 2020).

### Value Accruing to Society

In addition to value accruing to patients, antibiotics can generate elements of value that arise from externalities associated with antibiotic use and accrue to individuals other than patients. Therefore, it is important to quantify the value of an antibiotic at population level.

### Transmission Value

In the context of infectious diseases, society may attach value to the fact that successful treatment of an infected patient with an antibiotic may reduce or prevent transmission to other individuals in society. In an illustrative example of a hypothetical antibiotic to treat *Acinetobacter baumannii* infections, the incremental cost-effectiveness ratio of €36,570 per QALY gained in the standard economic evaluation improved to €4,318 per QALY gained in the economic evaluation that also accounted for transmission value. The health gain derived from avoided infections of 33,178 QALYs at population level surpassed the health gain accruing to patients of 12,442 QALYs (Morton et al., 2019). Another economic evaluation demonstrated that the inclusion of transmission value had a large positive impact on the cost-effectiveness of treating multidrug-resistant tuberculosis (Resch et al., 2006).

In order to account for transmission value, economic evaluation can be based on an epidemiological model that simulates the impact of the antibiotic on the dynamics of infection transmission in the population and computes the associated costs and health gains over time. As such models have been extensively used to include herd effects arising from vaccination, economic evaluation of an antibiotic can refer to relevant guidelines on modeling techniques such as those established by ISPOR’s “Economic Evaluation of Vaccines Designed to Prevent Infectious Disease: Good Practices Task Force” (Mauskopf et al., 2018). In the context of antibiotics, models need to account not only for infection transmission but also for resistance development (cfr. infra, Diversity value), and (dis)advantages of applicable modeling approaches (such as dynamic transmission models and statistical forecasting models) have been discussed in the literature (Rothery et al., 2018).

### Diversity Value

Antibiotic use is associated with resistance development (Chokshi et al., 2019), which imposes a significant health and economic burden on society. According to a simulation considering eight bacteria and 17 antibiotic-bacterium combinations for infections in five body sites, antimicrobial resistance in Belgium is on average associated with 4.6 deaths per 100,000 persons per annum; a loss of 114.1 DALYs per 100,000 persons per annum; and health care costs amounting to United States $ 240,397 per 100,000 persons per annum during the 2015–2050 period (Ouakrim et al., 2018). Economic evaluations considered resistance to the antibiotic under study by switching patients to another treatment or by including a cost and health loss due to resistance (Oppong et al., 2016; Kongnakorn et al., 2019a; Kongnakorn et al., 2019b). However, such studies did not include the impact that a new antibiotic may have on resistance levels to existing antibiotics that are replaced by the new antibiotic. A new antibiotic provides diversity value by decreasing the selection pressure on and the use of currently available antibiotics and, hence, reducing the development of resistance to these antibiotics.

The diversity value can be addressed by exploring the impact of various modes of employing new and existing antibiotics on antimicrobial resistance rates. These modes include the use of the new antibiotic as first-line treatment or treatment in a later or last line, use of new antibiotic dependent on pathogen diagnostic test results, rotating use among antibiotics, combined use of antibiotics, antibiotic stewardship programmes, etc. (Rothery et al., 2018). As each mode of employing antibiotics generates specific costs and health gains, incremental cost-effectiveness ratios of the alternative modes can be calculated, which provide insight in the diversity value.

Given that it is difficult to model the impact of an antibiotic on resistance development to other antibiotics, it has been argued that a formal expert elicitation exercise needs to be conducted to estimate the cost savings and health gains arising from lower selection pressure on these other antibiotics. This approach was followed in the previously mentioned example of a hypothetical antibiotic to treat *Acinetobacter baumannii* infections: the incremental cost-effectiveness ratio of the hypothetical antibiotic fell to €3,659 per QALY gained in the economic evaluation that also accounted for transmission and diversity values, and the health gain derived from avoiding resistant infections amounted to 2,752 QALYs (Morton et al., 2019).

### Insurance Value

In case a future catastrophic event (e.g., an exogenous increase in infections resistant to existing antibiotics) occurs, society may attach value to the availability of an effective antibiotic, which has been kept in reserve for use in this event. This can be exemplified by the availability of critical antibiotics for the treatment of secondary bacterial infections of COVID-19 patients. Insurance value consists of: 1) the conservation value associated with the strategy of not using the antibiotic until a catastrophic event arises; and 2) the precautionary value of having insurance against such an event (Neri et al., 2019). The former can be incorporated in economic evaluation by considering the use of the antibiotic as a last resort treatment. The latter can be investigated by eliciting the willingness to pay for avoiding a future catastrophic event or by exploring the impact of various antibiotic use strategies on the risk and health loss of this event. If society would attach more value to health outcomes from avoiding a catastrophic event than to other health outcomes (to be determined by means of public preference research), then a QALY adjustment factor needs to be taken into account (Rothery et al., 2018).

A recent study quantified the insurance value of withholding the use of a new oral antibiotic in the United Kingdom until the occurrence of pandemic influenza, which is associated with secondary bacterial infections, some of which are caused by a resistant *Staphylococcus aureus* strain (Megiddo et al., 2019). The authors found that the insurance value of this new antibiotic would amount to $2.2 billion and $578 million when 20% and 50% of patients, respectively, are treated intravenously during the pandemic. The insurance value increased when productivity loss due to mortality is considered. The insurance value was negative (which implies that it is better to use the antibiotic immediately) when the pandemic is mild and caused few infections with the resistant *Staphylococcus aureus* strain.

### Spectrum Value

There may be value related to the use of a narrow-spectrum antibiotic targeting a specific pathogen instead of a broad-spectrum antibiotic targeting a range of pathogens. This is because the use of broad-spectrum antibiotics may induce collateral damage to the microbiome, resulting in the development of future unrelated resistant pathogens (Neri et al., 2019). It is not clear if and how the spectrum value of antibiotics can be quantified. On the one hand, some authors have argued in favor of a qualitative assessment of the impact of various strategies of employing antibiotics on the development of resistance patterns and their associated costs and health outcomes (Rothery et al., 2018). On the other hand, given that diversity value, insurance value and spectrum value relate to (different aspects) of strategies of employing antibiotics, there is the risk of double counting when all these value elements are incorporated in economic evaluation (Neri et al., 2019).

### Novel Action Value

Novel action value refers to the value associated with an antibiotic that has a new mechanism of action. The innovative character *per se* of a health technology is of interest to reimbursement agencies, but does not tend to be valued separately. Indeed, it could be argued that the novel action value is actually captured by the costs and health gains generated by the antibiotic and its other value elements. Another aspect of novel action value, namely the fact that a new antibiotic may spur the development of follow-on antibiotics or other scientific advances, is difficult to quantify. Finally, within the specific context of antibiotics, the novel mechanism of action of an antibiotic is valuable in the light of preventing cross-resistance within existing classes of antibiotics (Karlsberg Schaffer et al., 2017).

### Enablement Value

Antibiotics may enable invasive surgical procedures or treatments for immunocompromized patients as their prophylactic use contributes to preventing and treating infections associated with these procedures. This enablement value can be quantified by exploring the impact of reduced efficacy or increased resistance to antibiotics on the number of infections and infection-related deaths associated with medical and surgical procedures. In a study focusing on antibiotic prophylaxis for the ten most frequent surgical procedures and immunosuppressing cancer chemotherapies in the United States, the authors found that a 30% decrease in the efficacy of antibiotics translates into an annual increase of 120,000 surgical site and chemotherapy-related infections and of 6,300 deaths (Ardal et al., 2018). Hence, it can be hypothesized that the enablement value of an antibiotic can be substantial.

## Aggregating Value Elements

Given that this discussion paper argues that antibiotics exhibit multiple elements of value, how can these be aggregated with a view to inform reimbursement decision making? A first approach is to “extend” standard economic evaluation and use modeling to examine the cost and health impact of different strategies of employing antibiotics on the spread of infections and resistance. This approach was adopted, for instance, in an economic evaluation of a new antibiotic to treat intensive care unit patients infected with *Acinetobacter baumannii* (Rothery et al., 2018). In another economic evaluation, transmission and diversity value was incorporated by adding related costs and health outcomes to the numerator and denominator, respectively, of the incremental cost-effectiveness ratio of the antibiotic (Morton et al., 2019).

A second approach relies on the technique of cost-benefit analysis and attaches a monetary value (by means of, for example, the willingness-to-pay method) to each value element with a view to quantify the benefit of an antibiotic. This approach has the advantage that all value elements can be aggregated in a single metric, but the feasibility and validity of measuring monetary benefits for each value element can be questioned. A third approach draws on multi-criteria decision analysis and assesses an antibiotic by calculating an overall weighted score of how it performs on the different value elements. Such an exercise requires that each value element can be quantified and that there is consensus on which value elements matter and what their relative weight is. To the best of the authors’ knowledge, no cost-benefit analysis or multi-criteria decision analysis calculating the aggregated value of an antibiotic has been published.

### Recommendations

**FIGURE 2:**
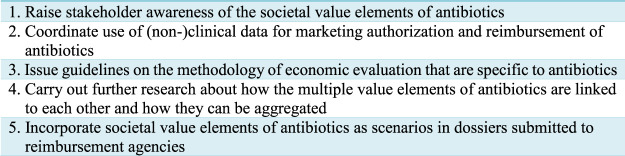
Recommendations to improve antibiotic assessment.

In order to move forward the debate about the value of antibiotics, we propose the following recommendations (see Figure 2).

First, there is a need to raise awareness and educate health economists, pharmaceutical companies, policy and decision makers that antibiotics are associated with multiple value elements and that economic evaluations need to be conducted at population level to account for externalities related to antibiotic use. This also implies that pricing and reimbursement of new antibiotics needs to reflect the societal value of antibiotics. Attention to the value of antibiotics needs to be paid in national action plans and strategies against antimicrobial resistance (European Commission, 2015) of individual European countries. Also, the ethical debate needs to be initiated about how different modes of employing new and existing antibiotics impact current patients vs. future patients in the light of resistance development (Leroy et al., 2019).

Second, regulatory authorities and reimbursement agencies need to consult each other with a view to streamlining the use of clinical and non-clinical data for the dual purposes of marketing authorization and reimbursement of antibiotics.

Third, reimbursement agencies need to issue methodological guidelines on how the standard economic evaluation framework can be modified to capture each of the different value elements of antibiotics. This could take the form of the approach followed by the Dutch Health Care Institute which provides guidance on whether and how other value elements of prevention, diagnostics, medical devices, long-term care and forensics can be considered in addition to the reference case of standard economic evaluation (Zorginstituut Nederland, 2016).

Fourth, in order to inform such guidelines, more research needs to be carried out on how to assess the societal value of antibiotics in general and on whether and how to quantify the spectrum value, novel action value and enablement value in particular. Also, further discussion is required about how the value elements of antibiotics can be aggregated, taking into account that value elements may be linked to or may overlap with each other (Neri et al., 2019). Moreover, future studies could apply techniques such as cost-benefit analysis or multi-criteria decision analysis with a view to calculating the aggregated value of an antibiotic.

Fifth, we advocate that economic evaluations of new antibiotics submitted to reimbursement agencies attempt to incorporate value elements of antibiotics accruing to society. These analyses can be presented as scenarios next to the calculation of the value accruing to patients. For instance, given that it is standard practice to apply modeling approaches to simulate the impact of vaccination on the dynamics of disease transmission, we need to learn from this experience with a view to account for transmission value of an antibiotic. Also, an economic evaluation needs to consider various modes of employing the new antibiotic, including its use as a last resort treatment.

## Conclusion

This discussion paper has argued that antibiotics are valuable to patients, but also generate value to society at large. This implies that standard economic evaluation under-values antibiotics if this broader impact is not considered. Therefore, we advocate that economic evaluation needs to account for not only the impact of antibiotic therapy on the infected patient, but also the impact of different modes of employing new and existing antibiotics on disease transmission and resistance development. Such an approach is required to maintain the value of antibiotics for current patients, for future patients and for enabling medical and surgical procedures that rely on antibiotic prophylaxis. An assessment of the societal value of antibiotics will also act as an incentive for pharmaceutical companies to invest in the research and development of new antibiotics. Finally, we recognize that consideration of the societal value of antibiotics raises the bar for those who carry out such economic evaluations and that relevant stakeholders (such as pharmaceutical companies and reimbursement agencies) need to go through a learning process to acquire the expertize and technical skills to conduct and assess economic evaluations demonstrating the societal value of new antibiotics. Due to resource and skill constraints, it may prove particularly challenging to overcome this hurdle in low- and middle-income countries.

## Author Contributions

SS developed the idea and design of this study, carried out the literature review and wrote the manuscript. IS critically reviewed the manuscript.

## Funding

The discussion paper was funded by Pharma.be, the Belgian industry association of innovator pharmaceutical companies.

## Conflict of Interest

SS has carried out health economic studies of antibiotics funded by Sanofi-Aventis, Bayer, TEVA, and the Belgian National Institute for Health and Disability Insurance. IS received unrestricted research grants from Pfizer and Merck, and travel support from Pfizer, Merck and Gilead.
